# Correction: Seledtsov et al. Inflammation Control and Immunotherapeutic Strategies in Comprehensive Cancer Treatment. *Metabolites* 2023, *13*, 123

**DOI:** 10.3390/metabo15100672

**Published:** 2025-10-15

**Authors:** Victor Ivanovich Seledtsov, Adas Darinskas, Alexei Von Delwig, Galina Victorovna Seledtsova

**Affiliations:** 1 Innovita Research Company, 06116 Vilnius, Lithuania; 2 Russian Scientific Center of Surgery Named after Academician B.V. Petrovsky, 119991 Moscow, Russia; 3 Institute for Fundamental and Clinical Immunology, 630099 Novosibirsk, Russia

**Removal Affiliation**

In the published publication [1], there was an error in the affiliation of Adas Darinskas. The affiliation National Cancer Institute, 08406 Vilnius, Lithuania, must be removed, and the correct affiliation is Innovita Research Company, 06116 Vilnius, Lithuania. The authors state that the scientific conclusions are unaffected. This correction was approved by the Academic Editor. The original publication has also been updated.
